# Biofilm stimulating activity of solanidine and Solasodine in *Pseudomonas aeruginosa*

**DOI:** 10.1186/s12866-023-02957-z

**Published:** 2023-08-02

**Authors:** Hadi Ghoomdost Noori, Omid Tadjrobehkar, Elham Moazamian

**Affiliations:** 1 Department of Microbiology, College of Sciences, Agriculture and Modern Technology, Shiraz Branch, Islamic Azad University, Shiraz, Iran; 2 Department of Medical Microbiology (Bacteriology and Virology), Afzalipour Faculty of Medicine, Kerman University of Medical Sciences, Kerman, Iran

## Abstract

**Background:**

Biofilm formation has reported as an important virulence associated properties of *Pseudomonas aeruginosa* that is regulated by quorum-sensing associated genes. Biofilm and quorum-sensing interfering properties of steroidal alkaloids, Solanidine and Solasodine were investigated in the present study.

**Results:**

Biofilm formation capacity and relative expression level of five studied genes(*lasI, lasR, rhlI, rhlR* and *algD*) were significantly increased dose-dependently after treatment with sub-inhibitory concentrations (32 and 512 µg/ml) of the both Solanidine and Solasodine. Biofilm formation capacity was more stimulated in weak biofilm formers(9 iaolates) in comparison to the strong biofilm producers(11 isolates). The *lasI* gene was the most induced QS-associated gene among five investigated genes.

**Conclusion:**

Biofilm inducing properties of the plants alkaloids and probably medicines derived from them has to be considered for revision of therapeutic guidelines. Investigating the biofilm stimulating properties of corticosteroids and other medicines that comes from plant alkaloids also strongly proposed.

## 1-Introduction

*Pseudomonas aeruginosa (P. aeruginosa)* is a Gram negative rod which has well known as a pathogen in both plants and animals [1]. *P. aeruginosa* is the causative agent of 7.1–9% of different nosocomial infections especially pneumoniae in in persons with compromised immune system and cystic fibrosis patients [2, 3]. *P. aeruginosa* is a multidrug resistant and biofilm former pathogen that classified as one member of the ESKAPE group which have introduced as the causative agents of severe nosocomial infections and major problem on the way to successful eradictional treatment [3, 4]. Mutations in the genes encoding antibiotic targeted components, acquisition of resistance associated genes from the environment, blocking penetration of antibiotics through structural changes in membrane and cell wall, production of antibiotic destructive enzymes, efflux pumps and finally biofilm formation are the major mechanisms that used by *P. aeruginosa* to resist antibiotic agents [5].

Biofilm formation is one of the important virulence associated factors that capable *P. aeruginosa* to effectively colonize and survive in the targeted sites. The biofilm matrix of *P. aeruginosa* composed a mixture of extracellular DNA, polysaccharides, proteins and lipids which encompasses the bacteria. This complex matrix protects bacterial cells from the environmental harsh conditions including host immune system, antibacterial agents and chemical warfare of other bacteria [6]. Bacterial cells within the biofilm population are synchronized with each other through a signaling network composed of special chemical agents that are known as quorum-sensing (QS) [7]. Four QS system were identified in *P. aeruginosa* including LasIR, Iqs, Pqs and RhlIR. All four QS regulons composed of so many genes, transcription of the genes in every QS system is regulated by some chemical autoinducers that are produced exclusively for each system. Las and Rhl are the most important QS systems and N-(3-oxododecanoyl) homoserine lactone and N-butyril homoserine lactone were introduced as the major autoinducer molecules in las and rhl systems respectively [8, 9]. QS associated genes also regulate production and release of so many virulence factors other than biofilm formation in *P. aeruginosa* such as pyocyanin, toxins, protease, elastase, motility, ion chelators, efflux pumps and expression of type 6 secretion system(T6SS) effectors [3, 10].

Disrupting of QS could be beneficial in several ways including inhibition of biofilm formation, virulence associated factors and antibiotic resistance, therefore QS interfering components are regarded as suitable therapeutic candidate [11]. In the recent decades, the great volume of studies were conducted to find such agents. In this regards, natural active ingredients of plants were broadly investigated [12–17]. Alkaloids are one major group of these agents which are provided by wide range of the plants, marine creatures and some amphibians. Versatile alkaloids are produced by plants as a part of defense mechanisms against pathogens [18].

Tomatidine, Solanidine (SN) and Solasodine (SS) are alkaloids which produced by *Solanaceae* family plants. The antibacterial, antifungal, antiviral, anticancer, anti-inflammatory and QS interfering properties of Tomatidine were reported formerly [19–23]. We surprisingly detect biofilm stimulating properties from sub-inhibitory concentrations of tomatidine in *P. aeruginosa*, recently [24]. But SN and SS were investigated more limited. In the present study we tried to investigate QS interfering properties of sub-inhibitory concentrations of Solanidine and Solasodine and also their effects on biofilm formation ability of *P. aeruginosa.*

## Results

### Minimal inhibitory concentration of the SN and SS

No inhibitory properties were detected from the Solanidine and Solasodine at the studied concentrations (125–2000 µg/mL).

### Quantitative analysis of biofilm formation

Biofilm formation ability of isolated bacteria were estimated based on the optical density (OD) of mature biofilm at 570 nm obtained from microtiter plate technique. Eleven out of 20 isolates showed an OD value more than four times of the ODc(sterile medium plus 1% glucose as control), these isolates were regarded as strong biofilm producer and 9 isolates have ODc < OD < )2 × ODc( and introduced as weak biofilm formers.

### quantitative analysis of biofilm formation after treatment with tobramycin, SN and SS

**Fig. 1:**
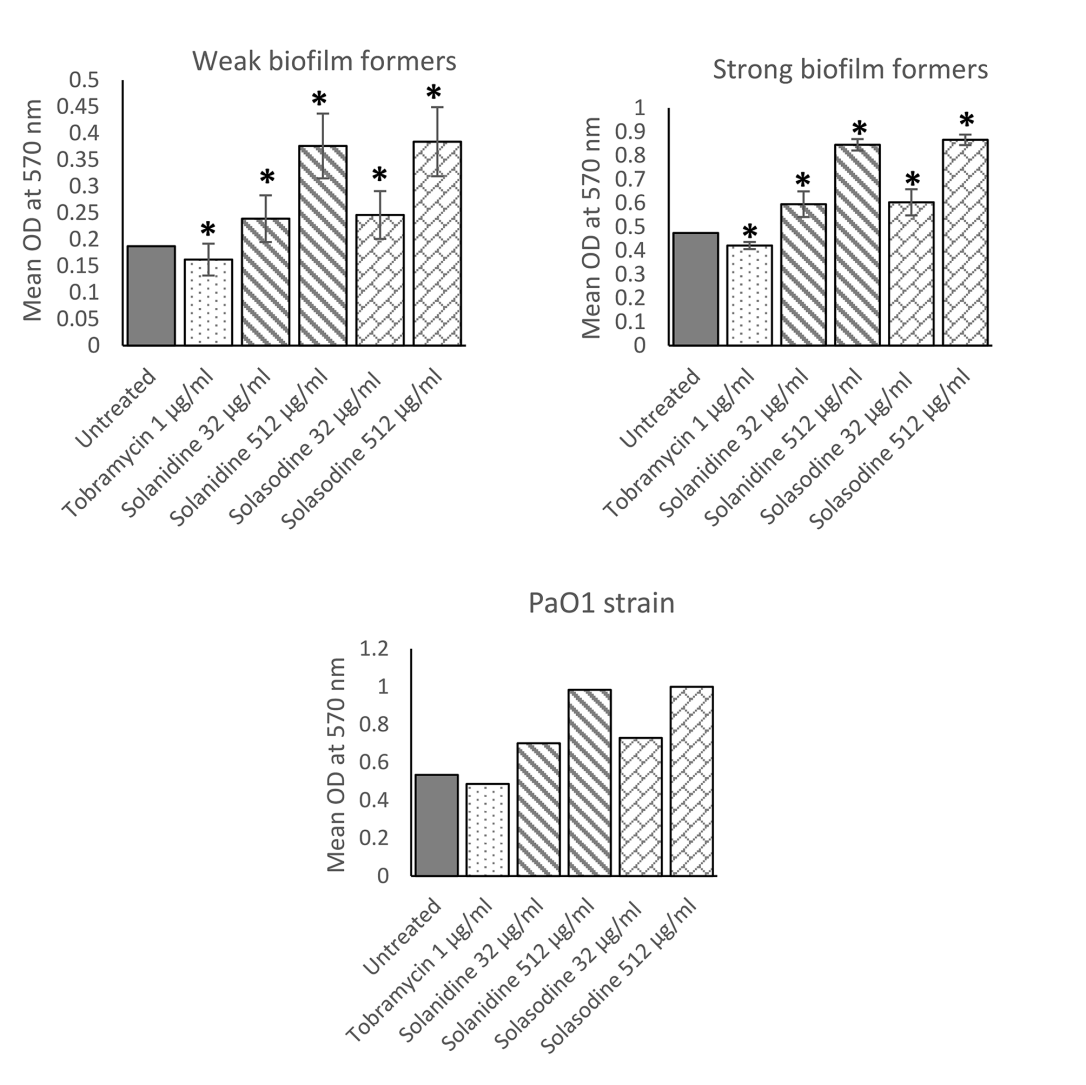
Biofilm inducing power(%) of 1 µg/ml tobramycin and two subinhibitory concentration (32 and 512 µg/ml) of SN and SS in comparison to the untreated condition as the control(red line) in weak biofilm formers and strong biofilm formers. Negative numbers showed biofilm reducing capacity

**Fig. 2:**
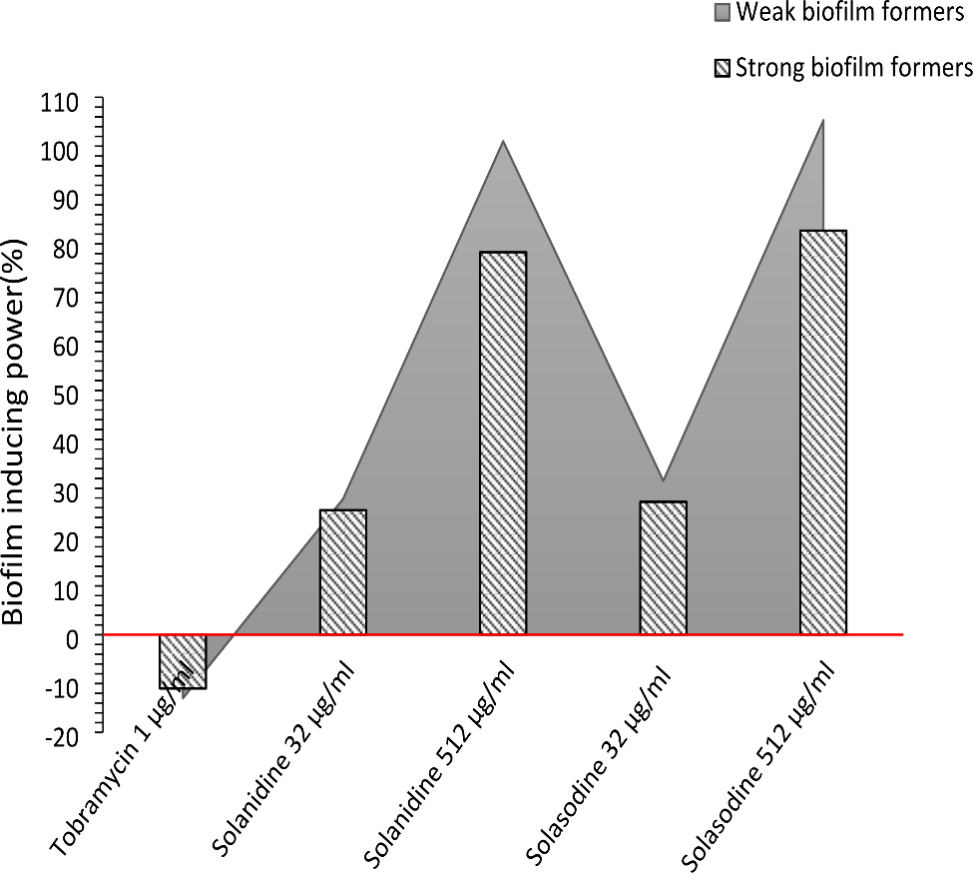
Quantitative analysis of biofilm formation (Mean optical density) of weak biofilm producers (9 isolates), strong biofilm producers (11 isolates) and PAO1 strain of *P. aeruginosa* in different treatment conditions. All treatment conditions(tobramycin 1 µg/ml and two concentrations of solanidine and solasodine) were compared with untreated condition as control. *Significant difference (*p* ≤ 0.05) was observed between the mean OD of treatment condition in comparison to untreated conditions

Biofilm production was reduced significantly(*p* = 0.001) after treatment with 1 µg/ml of tobramycin in comparison to untreated condition. Treatment with two sub-inhibitory concentrations(32 µg/ml and 512 µg/ml) of SN enhanced biofilm formation significantly in comparison to untreated condition in both weak biofilm formers and strong biofilm formers. Similar finding was observed concerning the SS. Treatment of isolates with sub-inhibitory concentrations(32 µg/ml and 512 µg/ml) of solasodine also enhanced biofilm formation significantly(Fig. 1). Statistical analysis also showed that induction of biofilm formation by the SN and SS is directly dose-dependent and higher concentrations of both studied alkaloids are significantly more potent inducer of biofilm formation in the both group of studied isolates in comparison to the lower concentration(Fig. 2).

Mathematical analysis showed that treatment with 1 µg/ml tobramycin reduced biofilm formation ability of the weak biofilm formers and strong biofilm formers, 13% and 11% respectively in comparison to the untreated condition. The 32 µg/ml concentration of SN enhanced biofilm formation ability of weak biofilm producers and strong biofilm producers 27.8% and 25.5% respectively, also 101% and 78.3% increased biofilm formation were detected in weak biofilm producers and strong biofilm producers after treatment with 512 µg/ml concentration of SN respectively. Similar phenomenon was detected after treatment with sub-inhibitory concentrations of SS, so that biofilm formation ability of weak biofilm formers and strong biofilm formers enhanced 31.5% and 27.2% after treatment with 32µ/ml and 105.3% and 82.7% after treatment with 512 µg/ml concentration of SS respectively(Fig. 1). There were not observed significant differences between biofilm inducing power of similar concentration of SN and SS in weak biofilm producers and strong biofilm producers(*p* ≤ 0.05).

### Relative expression level of biofilm associated genes in untreated condition in comparison to treatment with two sub-inhibitory concentrations of SN and SS

**Fig. 3:**
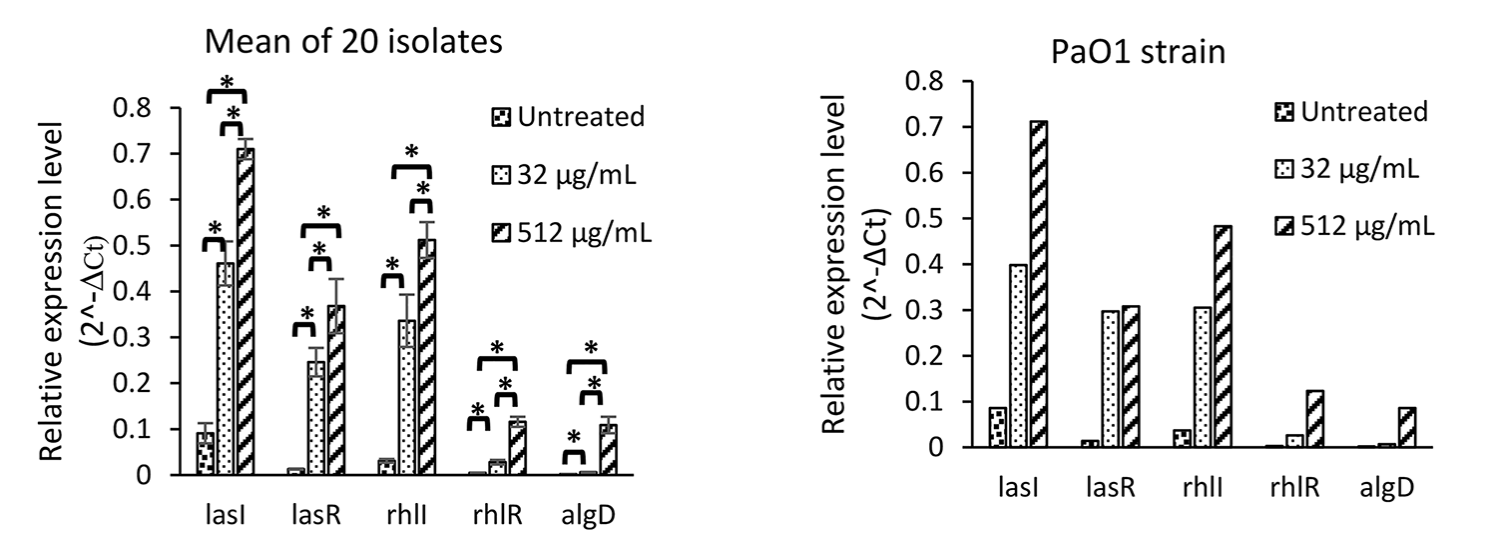
Relative expression level of five studied genes in untreated condition and in the presence of two sub-inhibitory concentrations (32 and 512 µg/mL) of Solanidine. *p ≤ 0.05

**Fig. 4:**
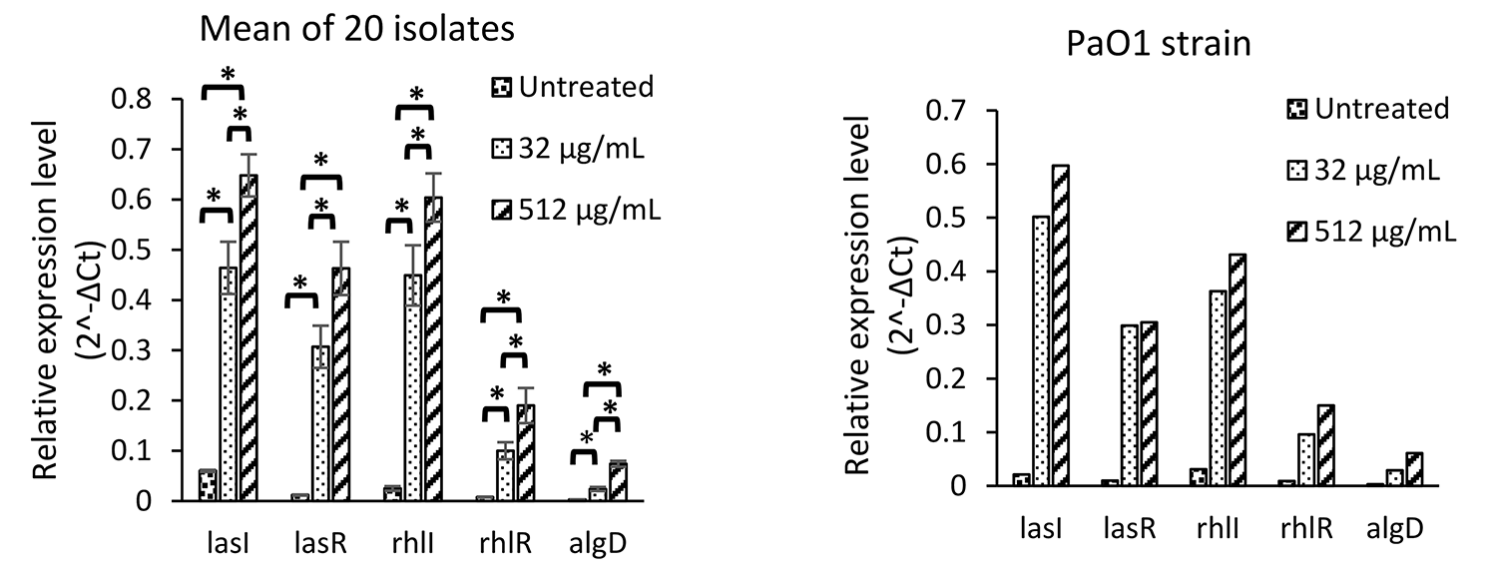
Relative expression level of five studied genes in untreated condition and in the presence of two sub-inhibitory concentrations (32 and 512 µg/ml) of Solasodine. *p ≤ 0.05

The paired t-test analysis revealed that, the mean expression levels of all five studied genes were increased significantly after treatment with two sub-inhibitory concentrations of SN (Fig. 3) and SS (Figs. 4) and 512 µg/mL concentration of both studied alkaloids was significantly more powerful inducer in comparison to 32 µg/mL concentration (Figs. 3 and 4).

### Relative expression level of the studied genes in weak biofilm formers in comparison to the strong biofilm formers in different conditions (untreated and two treatment condition)

**Fig. 5:**
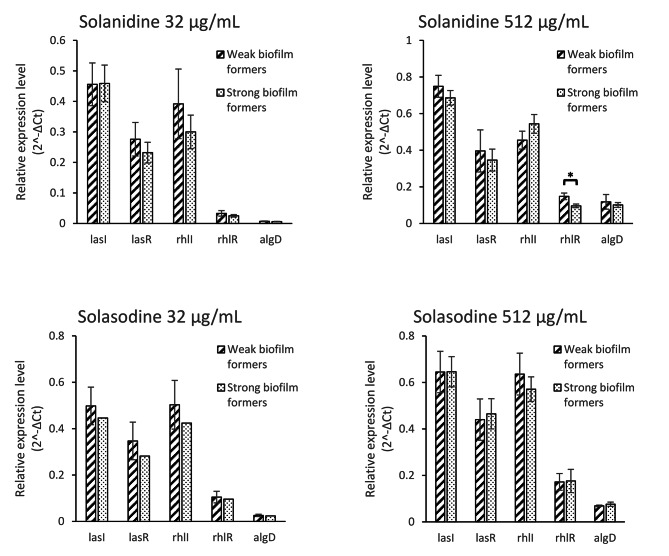
Relative expression level of five desired genes in weak biofilm formers in comparison to strong biofilm producers, after two treatment conditions (32 µg/mL and 512 µg/mL of SN and SS). *p ≤ 0.05

There was not observed significant differences between relative expression level of the studied genes in weak biofilm formers in comparison to strong biofilm formers after treatment with different concentration of SN and SS, except *rhlR* gene. Statistical analysis revealed that the relative expression level of *rhlR* gene increased significantly in weak biofilm formers in comparison to strong biofilm formers after treatment with 512 µg/ml of Solanidine (Fig. 5).

## Discussion

Antimicrobial properties of different natural products and plant-derived compounds such as alkaloid chemicals were reported in the recent years and they are also available commercially in medicine and traditional medicine [14, 25]. Although, antibiofilm and QS repressing activity of some herbal product were reported recently [26–30]. Dose-dependent biofilm inducing activity of sub-inhibitory concentrations of SN and SS were detected in the present study. Increased expression of some QSs associated genes also have seen in the experiments. Similar dose-dependent stimulation of biofilm formation in different bacterial species after treatment with subinhibitory concentration of antimicrobial agents such as aminoglycosides and fluroquinolones was reported recently [31, 32]. This phenomenon referred as Hormesis and liberation of biofilm structural blocks from the death cells and also development of stress responses against sub-inhibitory concentration of antimicrobial were hypothesized as the describing reasons [33].

In this study, we don’t detect any inhibitory properties from SN and SS until 2000 µg/mL concentration. While, antibacterial properties of some solanum alkaloids such as tomatidine and solasodine were reported against Gram-positive bacteria such as *Staphylococcus aureus*( *S. aureus*), *Bacillus* species and *Listeria* recently [20], Chagnon et al. were reported, medium activity of tomatidine(MIC > 64 µg/ml) against *S. aureus* but, they can’t detect any inhibitory properties from solanidine analogues. They also suggested that, spatial structure of the solanum molecule and specific orientation of active moieties have decisive role in their antibacterial properties, but no inhibitory activity from tomatidine and solasodine against small colony variant of *S. aureus* were detected [34]. The small colony variants are major bacterial morphology within biofilm [35]. In contrary, heavy antibacterial activity of tomatidine against small colony variant of *S. aureus* was reported by Boulet et al [19]. Thus, different spatial structure of different alkaloid molecules could be the main reason of different antibacterial potential of some alkaloid agents in comparison to the others.

Weak permeability of the Gram-negative bacteria due to the outer membrane layer and richer pool of the antimicrobial resistance mechanisms in Gram negatives such as *P. aeruginosa* in comparison to many Gram positives could explained less effectiveness of solanum molecules against them.

*P. aeruginosa* is usually known as an opportunistic pathogen for both plants and animals. But, some beneficial properties of this pathogen for plants; including promoting plant growth through antagonistic action against some bacterial and fungal phytopathogens and also promoting colonization of beneficial species were reported recently and critical leadership role of the QS systems especially LasIR and RhlIR have revealed in this process [36]. Therefore, secretion of some QS-inducing substances such as solanum chemicals or other alkaloids could be regarded as a plant inducing signal that enhanced expression of QS associated genes, biofilm formation and subsequently successful colonization of the beneficial bacteria. Our finding strongly supports this hypothesis.

The ATP synthase was introduced as the main target of tomatidine in *S. aureus* recently [19]. Therefore, different structure of bacterial targets such as ATP synthase in Gram positive and Gram negatives could be hypothesized as another reason. Ineffectiveness of some new antibiotics that knockdown ATP synthase in Gram positives, against Gram negatives was reported recently and support this hypothesis [37].

Biofilm inhibiting properties of 1 µg/ml tobramycin was detected in our study. Tobramycin also was reported as a biofilm reducing agent in other studies [38, 39]. Both SN and SS enhanced biofilm formation ability of *P. aeruginosa* isolates. Our finding showed that biofilm inducing properties of SN and SS was directly dose dependent and also both studied alkaloids were considered stronger biofilm inducer in weak biofilm producers in comparison to the strong biofilm producers(Figs. 2 and 1). We believe that strong biofilm producers have obtained heavy biofilm production ability and paid costs such as reduced competition characteristics, reduced virulence and probably irritability from environmental stresses, this phenomenon well reported as fitness cost in some recent studies [40].

The expression of all five investigated genes were increased significantly after treatment with both sub-inhibitory concentrations of SN and SS. The 512 µg/ml concentration of both studied alkaloid were more potent inducer in comparison to the 32 µg/ml concentration that support direct dose dependent inducing properties of solanum molecules. The *lasI* gene was the most induced gene by both SN and SS(Figs. 3 and 4). Therefore. It seems that the LasIR system is more important for biofilm formation in comparison to the RhlIR system as this is reported formerly in another study [41]. Regulation of RhlIR system by LasIR system and superiority of LasIR system over RhlIR system also reported formerly [10, 36].

Our finding revealed that the *algD* and *rhlR* were the least induced genes among the five studied genes. Statistical analysis also revealed that, the *rhlR* gene was significantly less expressed in weak biofilm producers in comparison to the strong biofilm producers after treatment with 512 µg/ml Solanidine (Fig. 5). Some other studies hypothesized that the RhlR in contrary to LasR is a major in vivo receptor that repress expression of some Qs associated virulence factors [42]. Thus, less involvement of *rhlR* gene in biofilm formation process and inducing QS associated mechanisms could be concluded.

Steroidal alkaloids derived from *Solanaceae* family such as Solasodine are employed as drug precursors in manufacturing many different storied drugs such as corticosteroids, antifertility drugs and steroid anabolic steroids [43, 44]. We also find some recent papers that reporting abolishment of antibacterial and antibiofilm activity of some antibiotic agents by anti-inflammatory corticosteroid drugs [45, 46]. Increased rate of treatment failure in combination therapy with antibiotics and corticosteroids also has reported previously [47]. Therefore, biofilm inducing properties of solanum alkaloids that was detected in the present study remind a warning about the same properties of solanum derived commercial drugs. Therefore, we propose to studying the biofilm inducing properties of such drugs specially in *invivo* condition and we believe that the acquired results could be beneficial and useful in revision of therapeutic prescriptions especially in biofilm associated infections and also therapy regiments containing combination therapy with antimicrobial agents and plant derived steroid drugs.

## Conclusion

Sub-inhibitory concentration of Solanidine and Solasodine stimulated biofilm formation in *P. aeruginosa* in a dose-dependent manner. They also have increased expression of LasIR and RhlIR QS associated genes vigorously. Biofilm inducing properties of the steroidal Phytoalkaloids and probably medicines derived from them has to be considered in development of new therapeutic regiments and also revision of existed guidelines. But, investigating the biofilm stimulating properties of corticosteroids and other medicines that comes from plant alkaloids on a larger group of different bacterial species is necessary to reach confirmation. Finally, we also believe that sub-inhibitory of solanum alkaloids could be used usefully in research field in direction to stimulate biofilm formation of the bacteria in culture media.

## Material and methods

### Bacterial isolates and phenotypic detection of biofilm formation

Twenty local isolates of *P. aeruginosa* were used in all assessments. Bacterial isolates were obtained from different clinical samples in a recent study [24]. PaO1 strain also was used in all experiments as a strong biofilm former control. Biofilm formation ability of the isolates was confirmed by microtiter plate method through detection the optical density of safranin-stained sessile bacteria on the bottom and the walls of the polystyrene wells at 570 nm. Biofilm former isolates were grouped as strong biofilm formers and weak biofilm formers according to the Stepanovic et al. method [48, 49]. All experiment were repeated in triplicate and investigated bacteria were preserved in -70 °C until use.

### Evaluation the minimum inhibitory concentration (MIC) of SN, SS and tobramycin

Broth microdilution method was used for evaluation the MIC of SN, SS( 125 to 2048 _µg/ml_, 1/2 serial dilution concentrations of both studied alkaloids were investigated) and tobramycin according to the CLSI guidelines [50]. MIC range of tobramycin among different isolates was 2–64 _µg/ml_. Therefor, 1 _µg/ml_ of tobramycin was used as control in the biofilm formation assessments.

### Investigation of the biofilm formation after treatment with tobramycin, SN and SS

Evaluation of the biofilm formation capacity of the studied isolates in the absence/presence of the subinhibitory concentration of SS, SN and tobramycin as control was the main goal. Therefore, all of the isolates were treated with the compounds at the start point of the experiment in the microplate wells. Biofilm formation ability of the studied isolates was investigated quantitively after treatment with 1 µg/ml of tobramycin and two concentration (32 and 512 µg/ml) of SN and SS in comparison to untreated condition. Mean optical density (OD) of resuspended stained mature biofilms at 570 nm was used in order to quantitative estimation of biofilm formation capacity of isolates(26).

### Evaluation the relative expression level of investigated genes (lasI, lasR, rhlI, rhlR and algD)

Quantitative real-time PCR (qPCR) was performed in order to evaluating relative expression level of investigated genes. Briefly, all studied bacteria were cultured in Luria-Bertani broth (Merck, Germany) in untreated and different treatment conditions as mentioned before, and incubated overnight at 37 °C. RNA was extracted from overnight cultures by RNA isolation kit (Dena-Zist, Iran). Purity and stability of the extracted RNAs was confirmed by calculation of the 260 nm/280 nm OD ratio of suspensions and electrophoresis on 1% agarose gel, respectively. The Viva 2-step RT-PCR kit (Vivantis, Malaysia) with Syber-Green master mix technology was used in synthesis of cDNA and amplification process. Step one plus Real-time PCR system (Marsiling industries, Singapore) and specific primers (Table 1) were used in qPCR amplification. The *gyrA* gene was used as internal reference gene [21]. The comparative CT method was used in calculation of the relative expression level (2^−ΔCT^) of the investigated genes in untreated and after different treatment conditions [51].

**Table 1:** List of the primers that were used in qPCR amplification process

Name	Forward primer (5’–3’)	Reverse primer (5’–3’)	Amplicon length (bp)	Tm (ºC)
*lasI*	CTTTTCCGACTGTACGCT	ATCATCTTCTCCACGCCT	108	57
*lasR*	CTTGGTTGACGGTTTTCT	CTCCACTCCAATTTTCCAC	54	57
*rhlI*	CAAACCCGCTACATCGTC	TGCACAGGTAGGCGAAGA	109	56
*rhlR*	GAGGCTTTTTGCTGTGGT	GGGTGAAGGGAATCGTGT	105	56
*algD*	GGGGCCAACAAGGAATACA	AGCACCAGCACATCGGAA	101	57
*gyrA*	ACACCGAGGCGAACAAGA	TAGTCGGTGGTGAGGAAGT	73	57

The specificity of the primers was evaluated by melt curves

### Statistical analysis

Statistical product and service solution (SPSS) software version 26 was used in data analysis. Mean OD of both groups of isolates(weak biofilm formers and strong biofilm formers) was compared in different treatment condition with untreated condition and with each other through analysis of variance (ANOVA). The mean expression level of investigated genes in untreated condition was compared with the same values in different treatment conditions by paired t-test analysis. The *p*-value ≤ 0.05 was regarded as significant.

## Data Availability

The datasets used and/or analyzed during the current study are available from the corresponding author on reasonable request.

## Abbreviations

*P. aeruginosa*: *Pseudomonas aeruginosa*
QS: Quorum-sensing
SN: Solanidine
SS: Solasodine
T6SS: Type 6 secretion system
OD: Optical density
MIC: Minimum inhibitory concentration
qPCR: Quantitative real-time PCR
SPSS: Statistical product and service solution software
ANOVA: Analysis of variance
